# *Lactobacillus crispatus* BC1 Biosurfactant Counteracts the Infectivity of *Chlamydia trachomatis* Elementary Bodies

**DOI:** 10.3390/microorganisms9050975

**Published:** 2021-04-30

**Authors:** Claudio Foschi, Carola Parolin, Barbara Giordani, Sara Morselli, Barbara Luppi, Beatrice Vitali, Antonella Marangoni

**Affiliations:** 1 Microbiology, DIMES, Alma Mater Studiorum, University of Bologna, 40138 Bologna, Italy; claudio.foschi2@unibo.it (C.F.); sara.morselli6@unibo.it (S.M.); antonella.marangoni@unibo.it (A.M.); 2 Department of Pharmacy and Biotechnology, Alma Mater Studiorum, University of Bologna, 40127 Bologna, Italy; barbara.giordani4@unibo.it (B.G.); barbara.luppi@unibo.it (B.L.); b.vitali@unibo.it (B.V.)

**Keywords:** lactobacilli, *Lactobacillus crispatus*, biosurfactants, *Chlamydia trachomatis*, fatty acids

## Abstract

Lactobacilli-derived biosurfactants (BS) have shown promising effects as antimicrobial molecules. Since *Lactobacillus crispatus* plays a crucial role in maintaining vaginal eubiosis, BS from this species could represent novel therapeutic agents to counteract sexually transmitted pathogens, such as *Chlamydia trachomatis* (CT). The aim of the present study was to assess the inhibitory effects of a BS produced by the vaginal strain *L. crispatus* BC1 on the infectivity of CT elementary bodies (EBs). For concentrations ranging between 1 and 0.5 mg/mL at 60-min contact time, *L. crispatus* BC1 BS displayed a highly significant anti-CT activity, with about 50% reduction of EB infectivity towards HeLa cells. To identify the components responsible for chlamydial inhibition, a panel of selected fatty acids, including those present in BS lipopeptidic structure, was tested against CT EBs. Pentadecanoic acid, myristic acid, β-hydroxy-myristic acid, and β-hydroxy-palmitic acid were able to significantly reduce EBs infectivity up to 5–0.5 µg/mL, concentrations that resulted to be non-toxic for HeLa cells. These data can contribute to the understanding of the biological role of lactobacilli in the vaginal niche, as well as to promote the application of their produced BS as an innovative and antibiotic-sparing anti-chlamydial strategy.

## 1. Introduction

The obligate intracellular bacterium *Chlamydia trachomatis* (CT) is the agent of the most common bacterial sexually transmitted infection (STI) worldwide [1]. In women, a high proportion of urogenital chlamydial infections are asymptomatic and thereby left untreated, favoring the occurrence of several complications and sequelae, including pelvic inflammatory disease, infertility, and ectopic pregnancy [2,3]. 

A vaginal microbiota dominated by lactobacilli is crucial for the prevention of several STIs, including *Chlamydia* [4,5,6,7,8]. The protective role of lactobacilli against urogenital pathogens is exerted through various mechanisms, such as the production of antimicrobial compounds (e.g., lactic acid, bacteriocins, hydrogen peroxide, biosurfactants), co-aggregation, competitive exclusion, and immunomodulation [9,10,11]. 

Until now, only a few studies have focused on the in vitro interaction between lactobacilli and CT, elucidating some of the antibacterial mechanisms displayed by lactobacilli [6,12,13,14]. Previous works demonstrated that lactate production and acidification of the environment are critical for the anti-chlamydial activity, in addition to the consumption of the carbonate source represented by glucose [6,12]. Moreover, lactobacilli can interfere with CT infection by altering lipid composition and α5 integrin subunit exposure in epithelial cells’ plasma membrane [14]. To the best of our knowledge, no studies have yet investigated the anti-chlamydial activity of biosurfactants derived from lactobacilli. 

Biosurfactants (BS) are amphiphilic compounds produced by microorganisms, anchored on the surface or secreted to the outside, with useful physicochemical and biological properties [15,16]. Among all their critical functions and applications, BS and particularly lactobacilli-derived BS have shown promising effects as antimicrobial molecules, both against bacteria and fungi [17,18,19,20]. Since *Lactobacillus crispatus* plays a crucial role in maintaining vaginal eubiosis [21], BS from this species could represent novel therapeutic agents to counteract CT infection. In this context, the aim of the present study was to assess the inhibitory effects of a BS produced by the vaginal strain *L. crispatus* BC1 on the infectivity of CT elementary bodies (EBs), as well as to identify the BS structural components related to the antibacterial activity. Our data can contribute to the understanding of the biological role of lactobacilli in the vaginal niche, as well as to promote the application of their isolated components against sexually transmitted pathogens. 

## 2. Materials and Methods

### 2.1. C. trachomatis Strain and Cell Culture

*C. trachomatis* strain GO/86, serotype D, was used for the experiments [6,22]. This strain was originally isolated from a patient with non-gonococcal urethritis and belongs to the collection of the Microbiology Laboratory of Sant’Orsola-Malpighi Hospital in Bologna (Italy).

HeLa cells (ATCC CCL-2), a cell line originated from a human cervix carcinoma, were grown to confluent monolayers in 5% CO_2_ at 37 °C. CT was propagated in HeLa cells, cultured in Dulbecco’s minimal essential medium (DMEM) (EuroClone, Pero, Italy), supplemented with 10% fetal bovine serum, 1% L-glutamine 200 mM, and antibiotics (vancomycin 10 mg/L, gentamicin 10 mg/L, and amphotericin B 0.3 mg/L). For the preparation of EBs, confluent HeLa cells were infected with CT in a DMEM medium supplemented with cycloheximide 1 μg/mL, centrifuged at 640× *g* for 2 h to facilitate cell penetration, then incubated at 37 °C with 5% CO_2_ for 48 h [13]. HeLa cells were then detached and fragmented by sonication, by using a Bandelin sonicator at minimum power. Samples were centrifuged at 500× *g* for 10 min at 4 °C, and supernatants, which contain EBs, were further centrifuged at 40,000× *g* at 4 °C for 1 h. The resulting pellets were re-suspended in sucrose-phosphate-glutamate (SPG) 0.2 M, divided into small aliquots, and stored at −80 °C. 

The infectious titre (inclusion-forming units (IFU)/mL) was assessed by immunofluorescence assay. Briefly, HeLa cells were infected with tenfold serial dilutions of bacterial stock, centrifuged at 640× *g* for 2 h, and incubated for 48 h at 37 °C. Afterwards, cells were fixed with methanol, and stained with a monoclonal antibody against the chlamydial membrane lipopolysaccharide antigen conjugated with fluorescein (Meridian, Cincinnati, OH, USA), as previously reported [22]. Slides were observed under epi-fluorescence microscope (Eclipse E600, Nikon, Japan) equipped with a super-high-pressure mercury lamp and Plan Fluor DLL 20×, 40×, 100× lenses. The total number of CT IFU was enumerated by counting all microscope fields.

### 2.2. Isolation of BS from L. crispatus BC1

*L. crispatus* BC1 was isolated from the vaginal swab of a healthy premenopausal woman [23]. BC1 strain was cultured in de Man, Rogosa and Sharpe (MRS) broth (Beckton, Dickinson and Co., Milan, Italy) supplemented with 0.05% L-cysteine, at 37 °C for 24 h in anaerobic jars containing GasPak EZ (Beckton, Dickinson and Co.). 

The cell-bound BS was isolated from *L. crispatus* BC1 as previously reported [19,24]. Briefly, BC1 strain was cultured in 1 L of MRS broth for 48 h in anaerobic conditions. The cell pellet was harvested by centrifugation (10,000× *g*, 15 min), washed twice in sterile distilled water, resuspended in 240 mL of sterile PBS, and gently stirred at room temperature for 2 h to release the cell-bound BS. The suspension was centrifuged, and the supernatant filtered through a 0.22 μm pore size filter (PES 0.22 μm syringe filters, VWR International, Milan, Italy). Cell-free supernatant was subjected to dialysis against demineralized water in a Cellu-Sep© membrane (molecular weight cut-off 6000–8000 Da; Spectra/Por 2 dialysis membrane, Spectrum Laboratories Inc., Rancho Dominguez, CA, USA) for 24 h at room temperature, and freeze-dried at 0.01 atm and −45 °C (Christ Freeze Dryer ALPHA 1–2, Milan, Italy). BS produced by *L. crispatus* BC1 possesses a lipopeptidic structure as defined by De Gregorio et al. 2020 [25]. 

### 2.3. Anti-Chlamydial Activity of BS

The antimicrobial activity of BS was tested against *C. trachomatis* EBs by in vitro inhibition assay. Lyophilized BS was solubilized in phosphate buffer saline (PBS) at a concentration of 2 mg/mL and serially diluted. The different concentrations of BS (ranging from 2 mg/mL to 0.12 mg/mL) were mixed with 5 × 10^3^, 5 × 10^4^, and 5 × 10^5^ CT EBs (100 µL of stock solutions of 5 × 10^4^, 5 × 10^5^, and 5 × 10^6^ IFU/mL, diluted in saline) in a final volume of 200 µL. The same amount of EBs mixed with PBS (in absence of BS) was used as control. Mixes were incubated for 7 and 60 min at 37 °C in 5% CO_2_ atmosphere. Afterwards, they were inoculated into HeLa cells, grown in DMEM medium to confluence in individual tubes containing sterile coverslips, and centrifuged at 640× *g* for 1 h to facilitate chlamydial cell penetration. At the end of the centrifugation, culture medium was replaced with fresh DMEM, and HeLa cells were incubated at 37 °C with 5% CO_2_ for 48 h. *C. trachomatis* infection was evaluated by counting chlamydial IFU by direct immunofluorescence as described above. The number of IFU was counted in 30 randomly chosen 200× microscopic fields. The pH values of the final solutions (BS added to CT EBs) were checked by a pH meter, after appropriate calibration. 

### 2.4. Anti-Chlamydial Effect of Selected Fatty Acids

The anti-chlamydial effect of selected fatty acids was assessed following the same protocol described for BS. In particular, tridecanoic acid, myristic acid, β-hydroxy-myristic acid, pentadecanoic acid, palmitic acid, and β-hydroxy-palmitic acid were purchased from CliniSciences (Rome, Italy), solubilized in DMSO, and serially diluted in PBS. The pH values of the final solutions (fatty acid solutions added to CT EBs) were measured by a pH meter. Fatty acids were tested against CT (5 × 10^3^ EBs) at final concentrations ranging from 50 to 0.05 µg/mL, after 60 min of incubation with EBs. 

### 2.5. Cytotoxicity of Fatty Acids

The cytotoxic effect of fatty acids on HeLa cells was assessed by 3-(45-dimethylthiazol-2-yl)-2,5-diphenyltetrazol (MTT) assay, as previously reported [25]. In particular, tridecanoic acid, myristic acid, β-hydroxy-myristic acid, pentadecanoic acid, palmitic acid, and β-hydroxy-palmitic acid were solubilized in DMSO (10 mg/mL) and diluted in DMEM complete medium immediately before cell treatment. HeLa cells were grown in 96-well plates to 70% confluence at 37 °C with 5% CO_2_, then treated with fatty acids at different concentrations, ranging from 50 to 0.05 µg/mL. The solubilizing agent (DMSO) at same dilutions was also tested and used as control, as well as untreated cells. After 24 h of treatment, medium was replaced by 110 μL of MTT solution in DMEM (final concentration 0.5 mg/mL) and the plates were further incubated for 4 h at 37 °C, 5% CO_2_. The formazan crystals that formed were dissolved by the addition of isopropanol and quantified by optical density at 570 nm using an EnSpire Multimode Plate Reader (PerkinElmer Inc., Waltham, MA, USA). 

### 2.6. Statistical Analysis

All statistical analyses were performed by using GraphPad Prism software (GraphPad Prism version 5.02 for Windows, GraphPad Software, San Diego, CA, USA, www.graphpad.com (accessed on 27 April 2021)). A one-way analysis of variance (ANOVA) test followed by Dunnett’s multiple comparison test was used to assess the anti-CT and cytotoxic effect of BS and selected fatty acids. Results were expressed as mean ± standard error of the mean (SEM). Statistical significance was determined at * *p* < 0.05, ** *p* < 0.01, and *** *p* < 0.0001. 

## 3. Results

### 3.1. Effect of L. crispatus BS against CT EBs

**Figure 1:**
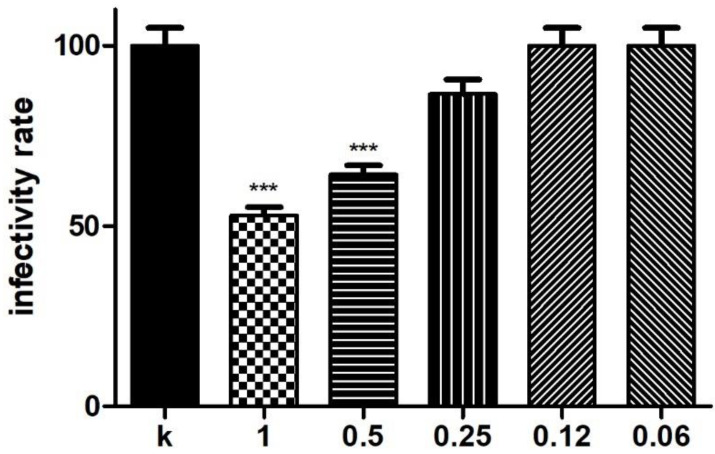
Anti-chlamydial effect of BS derived from *L. crispatus* BC1. Experiments were performed with different concentrations of BS diluted in PBS (final concentrations ranging from 1 to 0.06 mg/mL) added to 5 × 10^3^ EBs at 60-min time point. Chlamydial infectivity was evaluated as IFU/field and expressed as % of control (K) (5 × 10^3^ EBs incubated in PBS), taken as 100%. Bars represent mean values, error bars represent SEM. *** *p* < 0.0001.

BS isolated from *L. crispatus* BC1 displayed a significant killing activity against CT EBs (5 × 10^3^ EBs) for concentrations of 1 and 0.5 mg/mL after 60 min of incubation, as shown in Figure 1. Raw quantitative data on chlamydial IFU number, as well as images of HeLa monolayers infected with CT in the different experimental conditions, are shown in Supplementary Figure S1 and Table S1. Higher BS concentrations have not been tested against CT, considering the cytotoxic effect of BS on HeLa cells already demonstrated by De Gregorio et al. [25]. No inhibitory effect after 7 min of contact was noticed (data not shown). As expected, BS solutions maintained pH values of 7.0, thus excluding a killing effect due to an acidic milieu. 

When higher concentrations of CT EBs were used (i.e., 5 × 10^4^ and 5 × 10^5^ EBs), *L. crispatus* BC1-derived BS lost its anti-chlamydial activity (data not shown). 

### 3.2. Anti-Chlamydial Effect of Fatty Acids Contained in L. crispatus BC1-Derived BS

**Figure 2:**
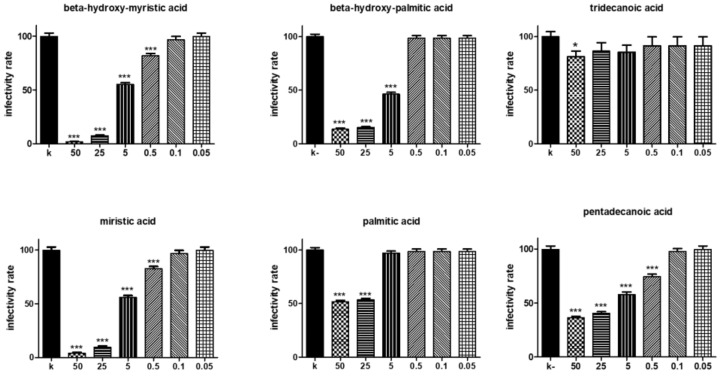
Anti-chlamydial effect of selected fatty acids. Experiments were performed with different concentrations of fatty acids (final concentrations ranging from 50 to 0.05 µg/mL) added to 5 × 10^3^ EBs at 60-min time point. Chlamydial infectivity was evaluated as IFU/field and expressed as % of control (K) (5 × 10^3^ EBs incubated in PBS), taken as 100%. Bars represent mean values, error bars represent SEM. *** *p* < 0.0001.

As previously described, the main fatty acids found in the BS were β-hydroxy-tridecanoic acid (3-OH-C13), β-hydroxy-tetradecanoic acid (3-OHC14; β-hydroxy-myristic acid), β-hydroxy-pentadecanoic acid (3-OH-C15), and β-hydroxy-hexadecanoic acid (3-OH-C16; β-hydroxy-palmitic acid) [25]. In line with these findings, to unravel the BS components associated with the antibacterial activity, we evaluated the anti-chlamydial effect of β-hydroxy-myristic acid and β-hydroxy-palmitic acid. Unfortunately, we were not able to test β-hydroxy-pentadecanoic acid and β-hydroxy-tridecanoic acids due to supply difficulties. In addition, we tested all the non-hydroxylated forms of the acids present in the lipopeptide structure (i.e., myristic acid, palmitic acid, tridecanoic acid, and pentadecanoic acid), in order to evaluate the effect of the beta substitution on biological activity (Figure 2). All the acids, except tridecanoic acid, displayed a high killing activity against CT up to concentrations of 5–0.5 µg/mL, with a dose-response effect. The best antimicrobial activity was exhibited by β-hydroxy-myristic and myristic acids, which were almost able to abolish CT viability at the highest concentrations tested. Interestingly, β-hydroxy-palmitic acid showed a much higher anti-chlamydial activity (50–60% more) compared to its non-hydroxylated form. The mixtures of CT EBs added with fatty acid solutions had pH values ranging between 7.4 and 7.5.

### 3.3. Cytotoxicity of Fatty Acids

**Figure 3:**
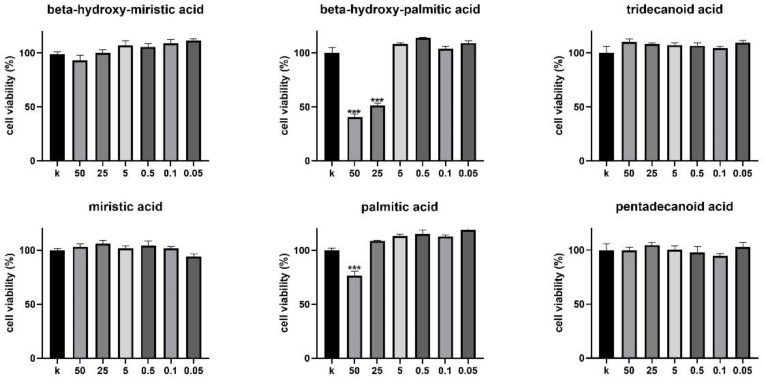
Cytotoxic effect of selected fatty acids on HeLa cells. MTT assay of HeLa cells treated with different concentrations of fatty acids (ranging from 50 to 0.05 µg/mL) for 24 h. Cell viability was expressed as % of control (K) (untreated cells), taken as 100%. Bars represent mean values, error bars represent SEM. *** *p* < 0.0001.

The same fatty acids tested on CT infectivity were also analyzed for their toxicity on cervical epithelial cells by MTT assay (Figure 3). β-hydroxy-myristic acid, miristic acid, tridecanoic acid, and pentadecanoic acid did not affect cell viability at concentrations up to 50 µg/mL. In contrast, palmitic acid and the corresponding hydroxylated form significantly decreased HeLa cells’ viability at the highest tested doses. In particular, 50 µg/mL β-hydroxy-palmitic acid reduced cell viability by 59.41%, whereas the same dose of palmitic acid reduced cell viability by 23.44%. β-hydroxy-palmitic acid induced a significant decrease of cell viability (48.71%) also when administered at 25 µg/mL. Vehicle molecule (DMSO) did not show any cytotoxic effect at the tested doses (data not shown).

## 4. Discussion

To the best of our knowledge, this is the first report assessing the activity of a *Lactobacillus*-derived BS against *C. trachomatis*. In particular, we investigated the anti-chlamydial effect of a BS isolated from a vaginal *L. crispatus* strain (*L. crispatus* BC1), by means of in vitro inhibition assays against CT EBs. For concentrations ranging between 1 and 0.5 mg/mL, *L. crispatus* BC1 BS displayed a highly significant anti-CT activity, with a 50% reduction of EB infectivity towards HeLa cells, when 5 × 10^3^ EBs were used. For higher chlamydial concentrations (5 × 10^4^ and 5 × 10^5^ EBs), *L. crispatus* BC1-derived BS lost its anti-chlamydial activity. In this context, it should be underlined that, usually, genital CT infections are characterized by low infectious and low bacterial loads [26,27]. Thus, the activity of lactobacilli BS against 5 × 10^3^ CT EBs better mimics the physiological and natural course of CT genital infections.

In previous works, we highlighted the antimicrobial activity of *L. crispatus* BC1 fractions (i.e., lactobacilli cells and cell-free supernatant) against several urogenital and STI pathogens, such as *Candida* spp., *C. trachomatis*, *Neisseria gonorrhoeae*, and *Streptococcus agalactiae* [6,7,8,23,28]. Moreover, *L. crispatus* BC1-derived BS was recently indicated as a promising safe agent able to interfere with important *Candida* virulence factors, i.e., adhesion to cervicovaginal cells and biofilm formation [20,25]. In this context, our data added knowledge on the functions of lactobacilli-isolated BS, shedding light on new antimicrobial mechanisms and opening the way to the use of BS as an innovative and antibiotic-sparing anti-chlamydial strategy.

Cell-bound BS recovered from *L. crispatus* BC1 were previously characterised from the chemical point of view and turned out to have a lipopeptidic nature [25]. To identify the BS components responsible for the inhibition of *Chlamydia* infectivity, a panel of selected fatty acids, including those present in *L. crispatus* BC1 BS structure, was tested against CT EBs. Such fatty acids were also assessed for safety on HeLa cells that represent the in vitro target of CT EBs and an appropriate cellular model in the perspective of using BS as a CT-infection antagonist.

Several long-chain fatty acids exerted a prominent killing activity against CT EBs, with a dose-dependent profile. In particular, β-hydroxy-myristic acid and β-hydroxy-palmitic acid, retrieved in *L. crispatus* BC1 BS structure, were able to significantly reduce EBs infectivity up to low concentrations. Besides this, anti-CT activity varied with the length of the fatty acid chain, with tridecanoic acid the less active and myristic acid the most active.

The effects of short chain fatty acids (SCFAs) produced by lactobacilli and other vaginal microbiota inhabitants (e.g., lactic acid, acetate, propionate, butyrate, and succinate) have been largely investigated, with the comprehension of their different antimicrobial and immune modulatory activities [29,30].

Conversely, the functionality of medium- and long-chain fatty acids on the microbial cells has been only partially explored. Medium- and long-chain free fatty acids have been found to have a broad spectrum of microbicidal in vitro activity against various bacteria, including some pathogens responsible for urogenital and sexually transmitted infections [31,32,33,34,35,36]. However, their antimicrobial effects varied greatly depending not only on fatty acid concentration and structure (length of fatty acid chain and type of derivatives), but also on the microorganism and bacterial species tested. In this context, Bergsson et al. showed that various fatty acids are able to inactivate *C. trachomatis* infectivity, by means of a direct disruption of EBs membranes [31]. For short contact times (i.e., 10 min of incubation), lauric acid, capric acid, and monocaprin acid cause a greater than 10,000-fold reduction in CT infectivity titre, whereas myristic acid, palmitoleic acid, and oleic acid have negligible effects on CT.

Here, we demonstrated that, after 60 min of incubation, myristic acid and its hydroxylated form are highly active against CT EBs, thus suggesting that contact time is a significant parameter to assess the antimicrobial effect of fatty acids. In addition, we proved, for the first time, the anti-chlamydial activity of pentadecanoic acid and β-hydroxy-palmitic acid. In our experience, palmitic acid exerted much less activity against CT EBs compared to its hydroxylated form. Interestingly, it has been shown that exogenous palmitic acid is incorporated into chlamydial synthesized phospholipids, thus potentially explaining the lower inhibition activity displayed by this acid compared to its hydroxylated derivative [37]. Overall, our data strengthen the idea that selected fatty acids may be useful as microbicidal compounds for the prevention and treatment of *C. trachomatis* infections [38].

*L. crispatus* BC1 BS was proven to be safe at doses capable of reducing CT infection [25]. In addition, among tested fatty acids, only β-hydroxy-palmitic acid and the respective non-hydroxylated form affected HeLa cells’ viability, suggesting a specific or preferential interaction of tetradecanoic fatty acid with epithelial cells. Although it is well recognized the induction of mitochondrial damage and apoptosis by free saturated fatty acids in multiple cell types, including hepatocytes and breast cancer cell lines [39,40], little is known on the contribution of fatty acid chain length on lipotoxicity. Indeed, several studies reported the use of palmitic acid or palmitate as the most representative saturated fatty acid, without analysing the effects of longer or shorter fatty acids [41,42]. Our results suggested a key impact of fatty acids chain lengths and hydroxylation on lipotoxicity.

In conclusion, this study elucidates the mechanisms underlying the protective role of vaginal microbiota against *C. trachomatis*, identifying BS and their acid components as important factors responsible for the inhibitory activity of lactobacilli. These results open the perspective of using both BS and the active fatty acids as new therapeutic agents as an alternative to traditional antibiotics that present problems of toxicity and bacterial resistance.

Further studies are needed to exactly quantify the amount of the various fatty acids in *L. crispatus* BC1 BS, as well as to understand the mechanisms of action by which fatty acids counteract EBs infectivity. In this context, ex vivo and in vivo models, such as polarized human fallopian tube epithelial cells and animal models [43,44], could be employed to better represent the complexity of CT infection and improve translational relevance.

## Data Availability

Data is contained within the article and Supplementary Material.

## Supplementary Materials

The following are available online at https://www.mdpi.com/article/10.3390/microorganisms9050975/s1, Figure S1: Images of HeLa monolayers infected with *C. trachomatis* in the different experimental conditions, Table S1: Raw data of *C. trachomatis* inclusion-forming units (IFU) number counted for each experimental condition.
