# Supplementary material for: Lactobacillus crispatus BC1 Biosurfactant Counteracts the Infectivity of Chlamydia trachomatis Elementary Bodies
Source: Microorganisms. 2021 Apr 30;9(5):975. doi: 10.3390/microorganisms9050975 (PMC8147163; doi:10.3390/microorganisms9050975)
Supplement: Supplementary file 1 [file microorganisms-09-00975-s001.zip › microorganisms-1164783-supplementary.pdf]

Supplementary Material

Lactobacillus crispatus BC1 biosurfactant counteracts the infectivity of *Chlamydia trachomatis* elementary bodies

Claudio Foschi, Carola Parolin*, Barbara Giordani, Sara Morselli, Barbara Luppi, Beatrice Vitali and Antonella Marangoni

Figure S1. Images of HeLa monolayers infected with *C. trachomatis* in the different experimental conditions. 5×10^3 *C. trachomatis* elementary bodies (EBs) were incubated with different concentrations of *L. crispatus* BC1 biosurfactant (BS) (final concentrations ranging from 1 to 0.06 mg/mL) for 60 minutes, then employed to infect HeLa cells. The images show parts of microscopic fields (magnification 200x) of cells stained with a monoclonal antibody against the chlamydial membrane lipopolysaccharide antigen conjugated with fluorescein. A: control (EBs with PBS); B-F: EBs with *L. crispatus* BC1 BS at a concentration of 1 mg/mL(B), 0.5 mg/mL (C), 0.25 mg/mL (D), 0.12 mg/mL (E), 0.06 mg/mL (F).

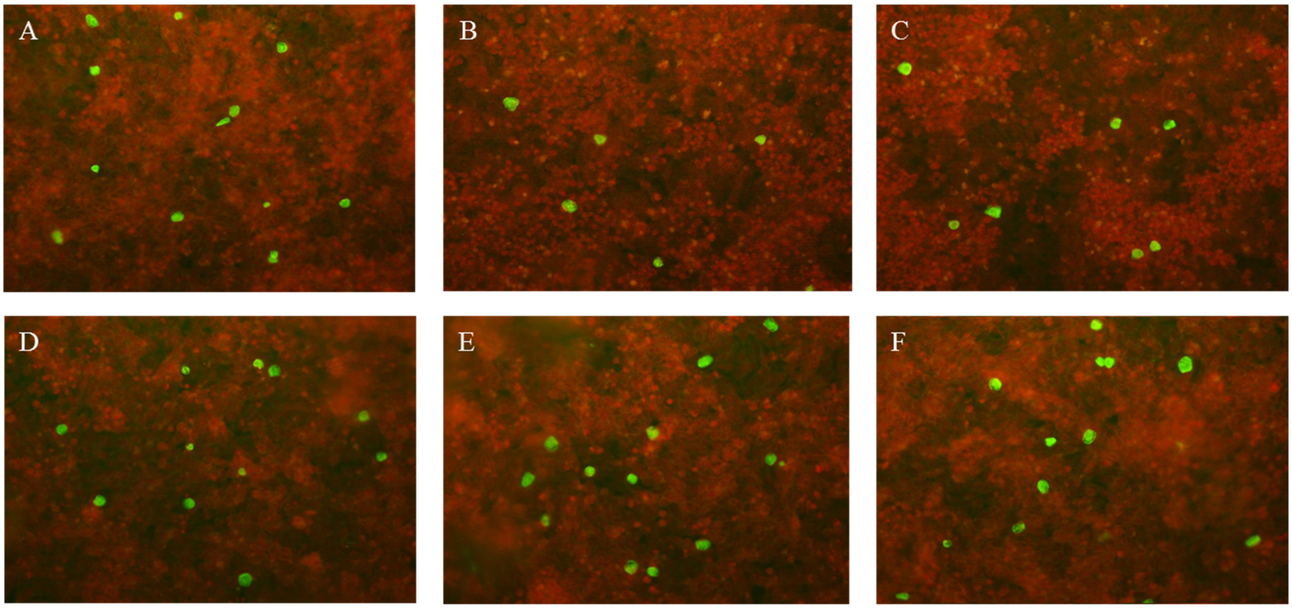

Table S1. Raw data of *C. trachomatis* inclusion-forming units (IFU) number counted for each experimental condition. 5×10^3 *C. trachomatis* EBs were incubated with different concentrations of *L. crispatus* BC1 BS (final concentrations ranging from 1 to 0.06 mg/mL) for 60 minutes, then employed to infect HeLa cells. Unconditioned EBs were used as control.

control	1 mg/ml BS	0.5 mg/mL BS	0.25 mg/mL BS	0.12 mg/mL BS	0.06 mg/mL BS
13	12	14	18	27	23
24	11	15	10	34	22
28	15	12	16	14	21
20	11	15	15	25	27
23	11	12	16	19	14
26	13	12	16	23	24
17	14	16	16	25	25
19	13	13	19	18	20
16	12	10	16	15	15
21	11	13	18	17	29
28	12	13	19	26	20
33	8	12	18	26	25
26	11	19	14	22	18
24	9	18	21	33	32
15	7	15	18	16	13
31	13	15	20	17	28
29	13	13	19	29	28
24	16	14	26	30	25
18	14	11	22	18	29
30	12	11	28	23	31
34	12	15	20	24	18
25	12	16	20	21	24
18	7	15	18	21	19
14	12	19	18	16	14
16	16	18	18	25	16
21	12	14	19	30	21
18	10	18	22	22	33
26	14	16	27	23	25
22	13	18	32	20	24
23	16	15	32	24	18
